# BIONOTE as an Innovative Biosensor for Measuring Endocannabinoid Levels

**DOI:** 10.3390/s21020489

**Published:** 2021-01-12

**Authors:** Simone Grasso, Marco Santonico, Giorgio Pennazza, Alessandro Zompanti, Alessandra Piccoli, Tiziana Bisogno, Mauro Maccarrone

**Affiliations:** 1 Department of Science and Technology for Humans and the Environment, Campus Bio-Medico University of Rome, 00128 Rome, Italy; s.grasso@unicampus.it (S.G.); m.santonico@unicampus.it (M.S.); 2 Department of Engineering, Campus Bio-Medico University of Rome, 00128 Rome, Italy; a.zompanti@unicampus.it; 3 Department of Medicine, Campus Bio-Medico University of Rome, 00128 Rome, Italy; ale.picc88@gmail.com; 4 Institute of Translational Pharmacology, National Research Council, 00133 Rome, Italy; tiziana.bisogno@ift.cnr.it; 5 Department of Biotechnological and Applied Clinical Sciences, University of L’Aquila, 67100 L’Aquila, Italy; 6 European Center for Brain Research, Santa Lucia Foundation IRCCS, 00143 Rome, Italy

**Keywords:** endocannabinoids, liquid biosensor, BIONOTE

## Abstract

In this study, a novel approach was developed to quantify endocannabinoids (eCBs), and was based on the liquid biosensor BIONOTE. This device is composed of a probe that can be immersed in a solution, and an electronic interface that can record a current related to the oxy-reductive reactions occurring in the sample. The two most representative members of eCBs have been analysed in vitro by BIONOTE: anandamide (*N*-arachidonoylethanolamine, AEA) and 2-arachidonoylglycerol (2-AG). Bovine serum albumin was used to functionalize the probe and improve the sensibility of the whole analytical system. We show that BIONOTE is able to detect both AEA and 2-AG at concentrations in the low nanomolar range, and to discriminate between these eCBs and their moieties arachidonic acid, ethanolamine and glycerol. Notably, BIONOTE distinguished these five different molecules, and it was also able to quantify AEA in human plasma. Although this is just a proof-of-concept study, we suggest BIONOTE as a cheap and user-friendly prototype sensor for high throughput quantitation of eCB content in biological matrices, with an apparent diagnostic potential for tomorrow’s medicine.

## 1. Introduction

The development of more and more sophisticated technologies and analytical techniques for the identification and quantitation of small molecules contributed to the study of lipid species, and to the understanding of their biological roles under health and disease conditions. Anandamide (*N*-arachidonoylethanolamine, AEA) and 2-arachidonylglycerol (2-AG) [1,2,3], two derivatives of the polyunsaturated fatty acid arachidonic acid (AA), were identified as endogenous ligands of the type-1 (CB_1_) and type-2 (CB_2_) cannabinoid receptors [4,5], and named endocannabinoids (eCBs) [6]. This fundamental breakthrough led to the identification of additional eCBs and eCB-binding receptors, as well as to *N*-acylphosphatidylethanolamine (NAPE)-specific phospholipase D [7] and diacylglycerol lipases α and β [8] as the main biosynthetic enzymes for AEA and 2-AG formation, respectively. The almost ubiquitous presence of the main eCB biosynthetic enzymes correlates with the role of these lipid modulators in multiple physiological processes. In particular, NAPE-PLD is highly expressed in brain, kidney, and testis [7] and DAGL in pancreas and brain [8]. Moreover, during brain development, DAGLα and DAGLβ are located in axonal tracts where they produce 2-AG and promote axonal growth and guidance, whereas both enzymes disappear from the growth cone and become postsynaptic in the adult brain, in order to support the release of 2-AG as retrograde messenger [8]. Moreover, fatty acid amide hydrolase [9] and monoacylglycerol lipase [10] were recognized as the main responsible for AEA and 2-AG hydrolysis to AA and ethanolamine or glycerol, respectively. Taken together, the signaling machinery that consists of eCBs, their receptor targets, and metabolic enzymes is known as the eCB system [11].

A wealth of results obtained from animal models and human studies over the past two decades provided evidence for an alteration of eCB tone in several pathological conditions, supporting the idea that targeting the eCB system could lead to therapeutic benefits, both at the periphery [12] and in the central nervous system [13]. Moreover, in the light of the fact that these changes of eCB levels occur in a strictly site- and time-specific way, eCBs have been widely recognized as useful biomarkers for prognostic/diagnostic purposes, able to predict disease onset and progression [12,13,14,15,16,17,18,19]. Evidence on animal models and humans highlighted the eCB crucial role as biomarkers of female infertility and their possible epigenetic effects on pregnancy [19]. Moreover, the modulation of eCB levels are associated with severity and progression of neurological disorders, including Parkinson’s disease, Alzheimer’s disease, Huntington’s disease, and multiple sclerosis [20]. Of note, a positive association of AEA with fasting insulin and insulin resistance has been also reported [21,22]. Thus, it seems apparent that to ascertain whether and how these lipid molecules are regulated under different physiopathological conditions, and to exploit changes of their content for diagnostic purposes, suitable methods for their accurate, precise and sensible detection and quantitation in cells, tissues, hair, nail and biological fluids are needed. Nuclear magnetic resonance spectroscopy and gas chromatography-mass spectrometry (GC-MS) were the key analytical techniques used to identify the chemical structure of AEA [1]. In the past few years, liquid chromatography (LC) coupled with MS (LC-MS) or tandem MS (LC-MS/MS) has become the reference procedure to detect and quantify eCBs in all biological samples [23].

It should be noted that these techniques are generally endowed with high-performance in terms of resolution, reproducibility and detection sensitivity; yet, they are also bulky, cumbersome, time-consuming and expensive, and require highly educated, trained and skilled operators. Thus, any device that could meet the criteria of affordability and user-friendly utilization, and that could be suitable for an industrial scaling-up and large market diffusion, might have a dramatic impact on scientific research of eCB signaling, and most notably on clinical routines for the diagnosis of eCB-related pathologies.

Biochemical detection is usually based on specific sensors functionalized with biomaterials, able to interact with specific molecules. To reach this goal, it is possible to distinguish two levels of design. The first level is the identification of a dedicated principle of transduction, for which it is mandatory to define specific parameters like resolution, sensitivity, and response time. These key parameters are addressed by the specific application. The transduction mechanisms can be very different: capacitive, piezoelectric, resistive or optical, just to cite the most used ones [24,25,26,27,28]. Each transducer has to be optimized with a dedicated electronic interface adapted to the application context, in order to counteract other confounding factors. In particular, when the response magnitude is close to the detection limit, a high gain of the front interface could help, but could also contribute to a noise increase that directly affects the sensitivity and, obviously, the resolution. These effects are more evident when specific power requests are mandatory, as in the case of energy harvesting systems [28]. The second level of design is related to the specific materials used as sensing elements that cover the transducer. For a proper biochemical detection, different materials could be used: organic, inorganic, or biological [29,30,31]. The optimization of deposited film has been largely studied [32,33,34] to obtain the best responses of the sensor. On the other hand, biosensors are designed to obtain a specific response to a specific molecule, but the system is often irreversible and consequently disposable. To avoid some of these problems, here we describe a novel approach based on a non-specific sensor supported by a dedicated electronic interface for the detection of lipids in solution. The system can use both functionalized and naïve sensors ensuring reproducibility and repeatability of the measurements. This sensor was conceived also for industrial applications to a fast detection of lipids. Of note, to the best of our knowledge this is the first time that such a multisensorial approach is developed for this type of application.

## 2. Materials and Methods

The calibration of the voltammetric sensor against authentic eCBs was performed by using AEA (A0580, Merck KGaA, Darmstadt, Germany), 2-AG (A8973, Merck KGaA, Darmstadt, Germany) and AA (23401, Merck KGaA, Darmstadt, Germany) standards. To test the discriminating performance of the analytical system against eCB moieties, glycerol (G5516, Merck KGaA, Darmstadt, Germany) and ethanolamine (ETA; 411000, Merck KGaA, Darmstadt, Germany) were chosen. Bovine serum albumin (BSA, fatty acid free; A7030, Merck KGaA, Darmstadt, Germany), ultra-low melting agarose (A2576, Merck KGaA, Darmstadt, Germany), and Tris Acetate-EDTA buffer (TAE; T8280, Merck KGaA, Darmstadt, Germany) were used for probe functionalization.

### 2.1. Voltammetric Sensor

The voltammetric sensor employed in the liquid analyses was described by Santonico and colleagues [35]. Briefly, the sensing platform is made of a Screen-Printed Electrode probe (SPE; DRP-250BT, Working: Gold, Counter: Platinum, Reference: Silver; Metrohm) and a dedicated electronic interface providing the input signal and recording the output data. When the SPE is immersed in a solution, an input signal consisting of a triangular waveform from +1 V to −1 V is applied and oxy-reduction phenomena involving the analytes dissolved in the aqueous media are induced. The current generated by the electrons involved in such reactions is captured by the system, and converted in voltage by a trans-impedance circuit. The frequency of the input signal was set to 0.01 Hz and for each measurement 500 samples were acquired. Five independent analytic cycles were run for each sample.

### 2.2. Data Analysis

Data analysis approach in this study treated the whole oxy-reductive profile provided by the samples as an electrochemical fingerprint. To get a simplified representation of the recorded multidimensional data set, multivariate data analysis techniques were applied. It should be recalled that partial least squares (PLS) regression is a statistical method that produces a bilinear factor model by projecting the predicted variables (Y) and the observable variables (X) to a new space. Partial least square discriminant analysis (PLS-DA) is a variant of PLS, that we have used in previous investigations [36,37], where the Y is categorical. PLS analysis aims at finding components which maximize the variability of predictors, showing at the same time the highest correlation with the response. To evaluate the reliability of a model, each sample dataset was separated into several different training and validation datasets, and the prediction accuracy metrics were averaged over all iterations. The cross-validation procedure checked whether the validation was similar among different training and validation datasets, and outputted the result as the root mean square error of cross-validation (RMESCV) parameter. All PLS analyses were performed using PLS-Toolbox (Eigenvector Research Inc., Manson, WA, USA) in the Matlab environment (The MathWorks, Natick, MA, USA), as we previously reported elsewhere [35,36,37].

### 2.3. Measure Setup

Stock solutions of eCBs were prepared by dissolving all standards in ethanol at the concentration of 500 µM, and then storing them at −20 °C until use. Working solutions were freshly prepared as needed by diluting eCB stock solutions in distilled water up to the concentration of interest (in the 100 µM to 0.001 µM range). Samples were stored on ice after their preparation, and were analyzed without further modifications. Voltammetric measurements were performed following two slightly different setups according to the type of SPE employed: the analysis with unmodified electrodes started immediately after their sinking, while functionalized SPEs were left to soak for 5 min in sample solution before triggering sample electrochemical stimulation.

### 2.4. SPE Functionalization

Briefly, the procedure to modify the gold working electrode was as follows. Bovine serum albumin (BSA) solution at the concentration of 200 mg/mL was prepared in distilled water, and was mixed with a hot 2% agarose solution (prepared in TAE or distilled water) with a volume ratio of 1:1. Ten µL of the prepared mixture was finally dispensed by drop casting onto the surface of the SPE working electrode, and was let to air dry until complete dehydration.

### 2.5. Lipid Extraction from Human Plasma and AEA Analysis

Human plasma was obtained from healthy donors recruited at Santa Lucia Foundation, who gave their written informed consent according to the Legislative Decree n. 196/2003. The study was approved by the Ethical Committee of Santa Lucia Foundation (protocol CE/Prog. 589, 23.01.2017), and was conducted according to the ethical principles arising from the Helsinki Declaration. Samples were extracted in chloroform/methanol/50 mM Tris–HCl (2:1:1) containing d8-AEA and d5-2-AG (Cayman Chemicals, Ann Arbor, MI) as internal standards. The lipids-containing organic phase was pre-purified by open bed chromatography on silica, and then aliquots of fractions eluted with 90:10 (*v*/*v*) chloroform/methanol, containing *N*-aylethanolamines and monoacylglycerols, were analyzed by LC–MS. LC-atmospheric pressure chemical ionization–MS investigation was carried out by using a Shimadzu high-performance liquid chromatography apparatus (LC-10ADVP), coupled to a Shimadzu (LCMS-2020) quadrupole mass spectrometry. LC analysis was performed in the isocratic mode using a Discovery C18 column (15 cm × 4.6 mm, 5 μm) and methanol/water/acetic acid (85:15:0.1 by vol.) as mobile phase with a flow rate of 1ml/min. Temperature of ionization source was 400 °C. MS detection was performed in selected ion monitoring (SIM) mode of positive ions by using m/z values of 356 and 348 (molecular ions +1 for d_8_-AEA and AEA) and 384 and 379 (molecular ion +1 for d_5_-2-AG and 2-AG). Amounts of AEA and 2-AG expressed as picomoles were then normalized per ml of plasma.

## 3. Results

### 3.1. Setup Optimization

**Figure 1:**
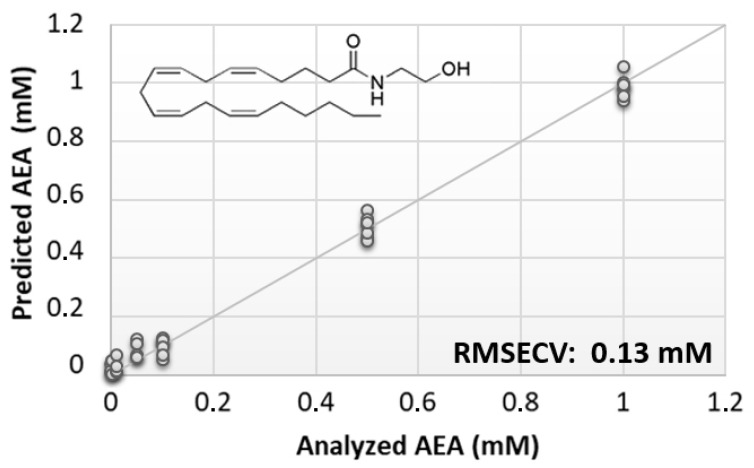
Calculated PLS-DA model for the prediction of *N*-arachidonylethanolamine (AEA) using unmodified SPEs.

The preliminary phase of this study was aimed at evaluating the feasibility of the BIONOTE device coupled with unmodified SPE to analyze eCBs. From the rather large family of these lipids, AEA was selected due to its prominent biological activity [38,39]. Aqueous solutions of AEA standards, at the concentrations of 1, 0.500, 0.100, 0.050, 0.010, 0.005, and 0.001 mM, were analyzed with the voltammetric sensor to test the ability of the instrument to quantitatively detect these compounds in liquid media. The PLS regression model calculated on the experimental data set, using the leave one out criterion, showed a root mean square error of cross validation (RMSECV) of ~130 µM (Figure 1).

Since the sub-millimolar error associated with the obtained model was too large to allow detection of eCBs at physiological nanomolar levels, sensor functionalization was exploited to enhance BIONOTE performance. To this end, bovine serum albumin (BSA) was chosen as a suitable biochemical material for SPE functionalization, because of its ability to bind circulating lipids in blood, and in particular eCBs with high affinity [38].

**Figure 2:**
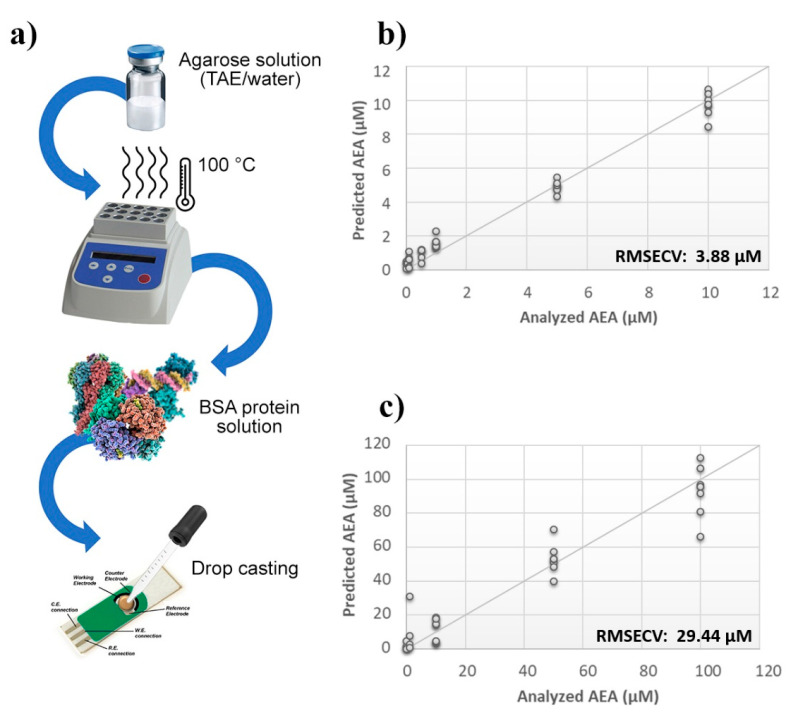
Evaluation of SPEs functionalization. (**a**) Schematic representation of SPE functionalization procedure. Calculated PLS-DA model for the prediction of *N*-arachidonylethanolamine (AEA) using (**b**) aqueous BSA-agarose or (**c**) TAE BSA-agarose functionalized SPEs.

A thin film of TAE or aqueous agarose gel containing BSA was deposited by a drop casting technique onto the surface of the probes, and then sensors were challenged against AEA standard solutions (Figure 2a). The calculated PLS model highlighted a considerable improvement of the BIONOTE sensibility over the unmodified SPE. However, while the aqueous BSA-agarose film allowed to decrease the RMSECV up to ~4 µM, the TAE BSA-agarose functionalization retained an error of ~30 µM (Figure 2b,c). Therefore, to further tune the detection capability of the device, modifications in the frequency of the electrochemical input were tested. Aqueous solutions of AEA standards at concentrations ranging from 1 µM to 1 nM were analyzed through the BIONOTE, coupled with aqueous BSA-agarose functionalized SPE employing cyclic voltammetry at 0.01 Hz and 0.1 Hz, independently.

**Figure 3:**
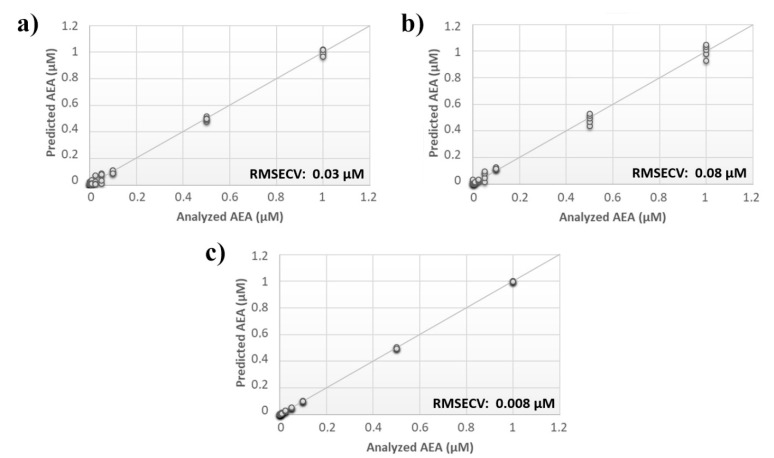
Evaluation of multiple input frequency analysis. Calculated PLS-DA model for the prediction of *N*-arachidonylethanolamine (AEA) using aqueous BSA-agarose functionalized SPEs applying an input frequency of (**a**) 0.01 Hz or (**b**) 0.1 Hz and (**c**) the data fusion of both measurements.

A comprehensive array containing the overall responses of sensors from both analyses was built, and the calculated PLS model was compared with the models originating from the individual data sets. Although the RMSECV for the single analysis was ~30 nM and ~80 nM for the input frequency of 0.01 Hz and 0.1 Hz, respectively, the error associated with the data fusion was substantially decreased to 8 nM (Figure 3).

### 3.2. Analysis of Endocannabinoids and Their Chemical Moieties

**Figure 4:**
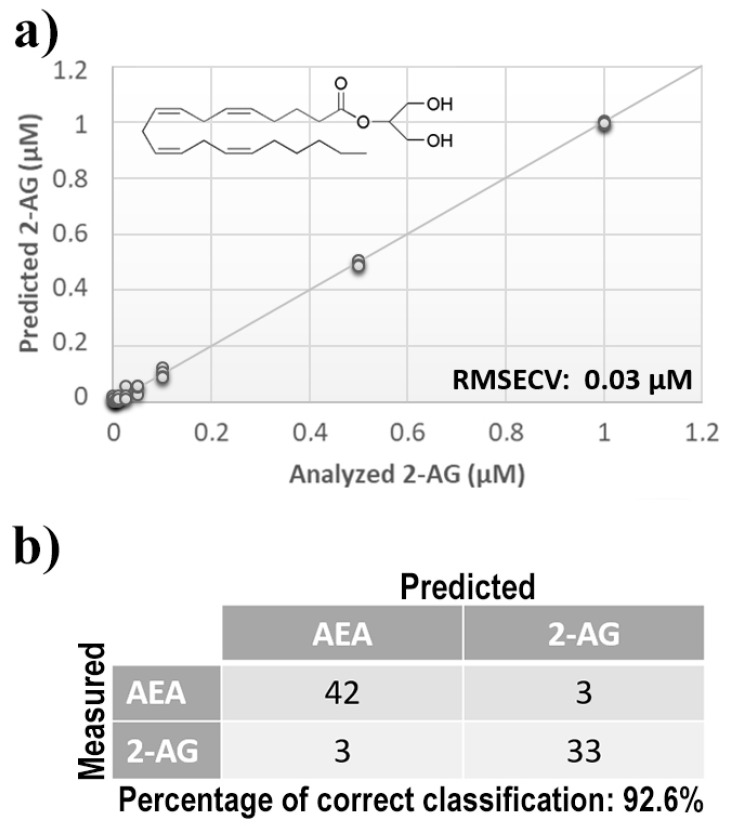
(**a**) Calculated PLS-DA model for the prediction of 2-arachidonoylglycerol (2-AG) using aqueous BSA-agarose functionalized SPEs and 0.01–0.1 Hz data fusion. (**b**) PLS-DA classification model for AEA and 2-AG.

**Figure 5:**
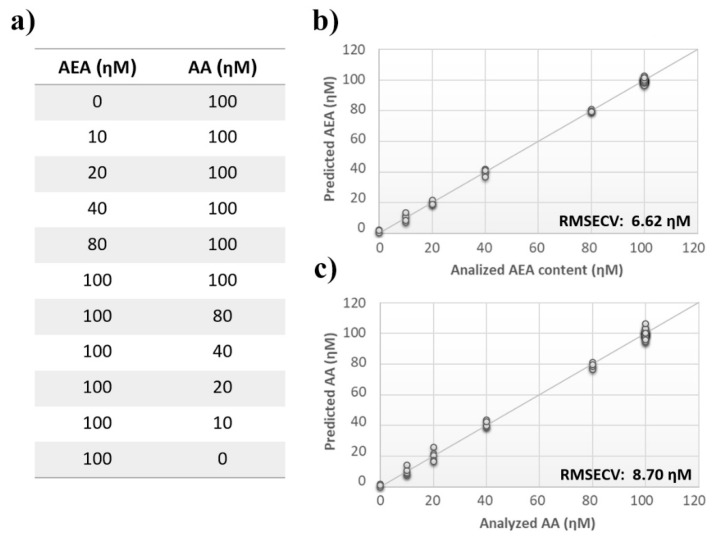
Analysis of multiple ECs standard mixture. (**a**) ECs’ measurement schema of the experimental setup. (**b**) Calculated PLS-DA model for the prediction of *N*-arachidonylethanolamine (AEA) using aqueous BSA-agarose functionalized SPEs and 0.01–0.1 Hz data fusion. (**c**) Calculated PLS-DA model for the prediction of arachidonic acid (AA) using aqueous BSA-agarose functionalized SPEs and 0.01–0.1 Hz data fusion.

**Figure 6:**
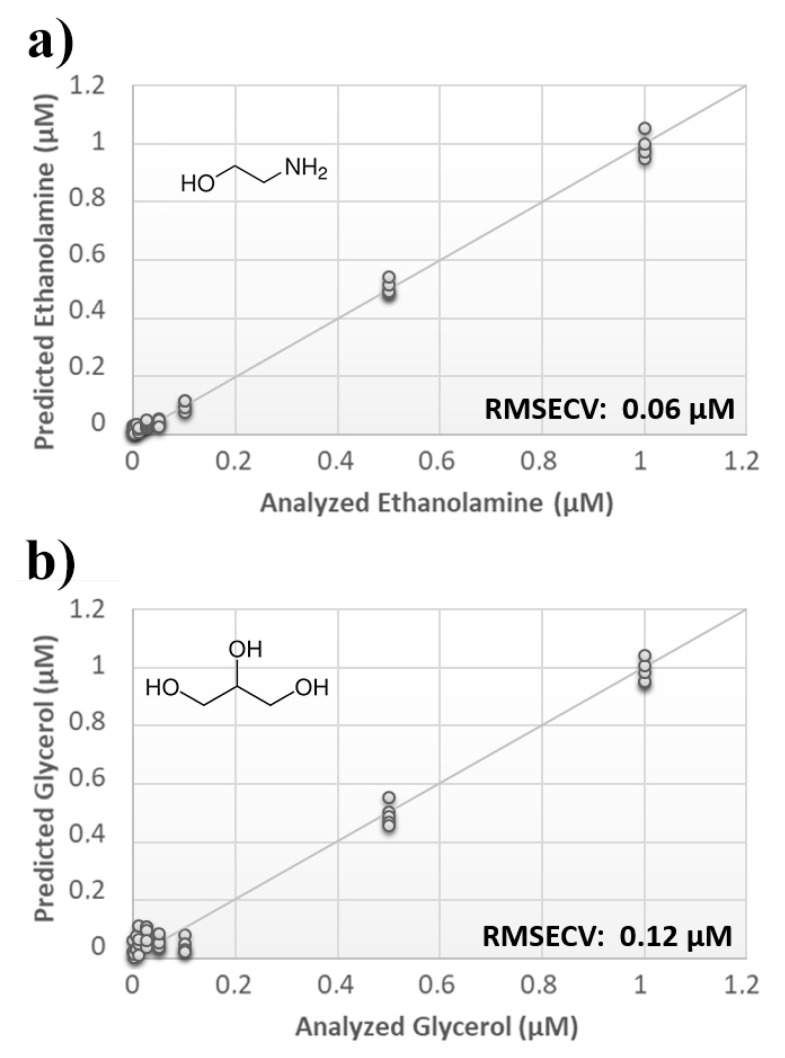
(**a**) Calculated PLS-DA model for the prediction of ethanolamine using aqueous BSA-agarose functionalized SPEs and 0.01–0.1 Hz data fusion. (**b**) Calculated PLS-DA model for the prediction of glycerol using aqueous BSA-agarose functionalized SPEs and 0.01–0.1 Hz data fusion.

**Figure 7:**
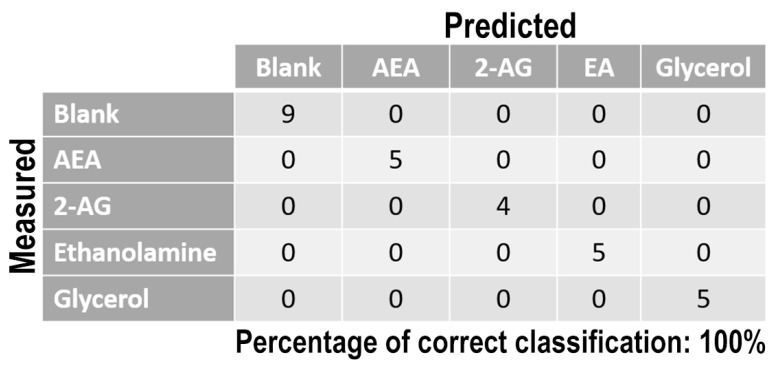
PLS-DA classification model for the lowest ECs and ECs-related standard concentration tested. Blank samples represented the aqueous solution without ECs molecules.

Once measurement conditions were optimized and AEA analyses were concluded, the other eCB with prominent biological activity, 2-AG [38,39], was used to extend BIONOTE calibration against eCB compounds. Aqueous solutions of 2-AG standards, at the concentrations of 1, 0.5, 0.1, 0.05, 0.025, 0.01, 0.05, and 0.001 µM, were analyzed with the voltammetric sensor and the obtained results were elaborated through multivariate data analysis. The PLS regression model calculated on the experimental data sets, using the Leave One Out criterion, showed a RMSECV of ~30 nM (Figure 4a). Next, the whole data collected from AEA and 2-AG calibrations were merged in one array, and a discriminating model was calculated. Remarkably, the computed PLS-DA model was able to distinguish the electrochemical fingerprints of the two eCBs with an efficiency in the classification of ~93% (Figure 4b). Afterwards, in order to evaluate the ability of the system to discriminate the presence of multiple compounds in the same solution, mixtures containing different concentrations of AEA and its fatty acid component AA were prepared. Eleven combinations of the two compounds were analyzed following the optimized measurement setup (Figure 5a), and the obtained data were processed through multivariate data analysis. Also, under these conditions, BIONOTE was able to detect both AEA and AA, with a maximum RMSECV of ~9 nM (Figure 5b,c). Finally, the voltammetric sensor was challenged against the two hydrophylic moieties of AEA and 2-AG: ethanolamine and glycerol, respectively. Aqueous solutions of standards of these molecules, at the concentrations of 1, 0.5, 0.1, 0.05, 0.025, 0.01, 0.05, and 0.001 µM, were prepared and then independently analyzed by the liquid sensor. Surprisingly, the PLS model calculated on the collected data highlighted a remarkable decrease of system performance in the detection and quantitation of ethanolamine and glycerol, with an obtained RMSECV of ~60 nM and ~120 nM, respectively (Figure 6). Thus, a comprehensive data set was built merging the results from each individual analysis of AEA, 2-AG, ethanolamine, and glycerol at the lowest concentration tested, and a discrimination model was calculated. Remarkably, the new PLS-DA analysis demonstrated the ability of the system to fully distinguish the fourmolecules, with an efficiency of ~100% in the classification (Figure 7).

Our next goal was to test the BIONOTE performance in detecting AEA in an authentic biological matrix. To this end, lipids were extracted from human plasma, and the pre-purified fraction containing AEA was analyzed with the voltammetric sensor. The resulting electrochemical signal was given as input to the predicting model built on AEA standard. Moreover, AEA quantitation by the BIONOTE device was compared with that obtained from the same samples by a classic LC-MS analysis. It was found that AEA concentration in human plasma predicted by BIONOTE was ~34 pmol/mL, whereas that detected by LC-MS was ~10-fold lower (3.85 ± 0.32 pmol/mL; s.d.). As expected, BIONOTE efficiently discriminated the electrochemical fingerprints of AEA vs. 2-AG, yet the RMSECV associated to the latter eCB did not allow an accurate metabolite quantification, and hence a comparison with the concentration assessed by LC-MS analysis.

## 4. Discussion

In this investigation we report the further development of an innovative lipid biosensor, able to assess eCB content in liquid media with a reasonable accuracy. In a previous work [40] the feasibility of this technique was proposed, showing good results based on preliminary data. In this work the biosensor was tested and calibrated in order to demonstrate that our voltammetric system was able to quantify pure AEA, 2-AG, and AA with a nanomolar error in calibration procedures. The obtained results demonstrate that electrode functionalization by the eCB-binding protein BSA, coupled with a multi-frequency analysis, was able to considerably improve device sensibility towards eCBs, when compared with unmodified SPEs. Interestingly, such enhanced sensitivity appeared to be specific for eCBs detection. Indeed, when moieties of eCBs were analyzed by the BIONOTE device, a lower performance was observed with 10-fold higher errors.

To the best of our knowledge, this is the first time that a lipid sensor like BIONOTE has been developed to discriminate the electrochemical fingerprints of eCBs in vitro and, more importantly, in lipid fractions extracted from authentic biological samples like human plasma.

Despite electrochemical fingerprinting of eCBs can be used for their identification, eCB levels predicted by BIONOTE were ~10-fold higher than those detected by chemical fingerprinting provided by LC-MS, suggesting that the new lipid sensor needs indeed to be further implemented to become widely applied for sensible detection and quantitation of eCBs.

It should be noted that eCB quantitation in biological matrices is a rather challenging task, because of the complexity of the samples and of the analytical procedures required to optimize the whole process (e.g., sample preparation, extraction, chromatographic separation, and detection). In particular, liquid-liquid extraction (LLE) or its salting-out variation [41], solid phase extraction (SPE) and µ-SPE tips [42,43], as well as column switching techniques [44], are only same of the procedures presently used to improve both lipid extraction and the long clean-up processes necessary for partial or full sample purification. Moreover, it should be appreciated that currently available analytical procedures for eCB quantitation in the pmol/mL (i.e., nM) range have required many years of methodological developments, to warrant high enough sensitivity and selectivity with reduced sample size and derivatization, high-performance liquid chromatography (HPLC) and ultra-HPLC methods, with subsequent coupling to different MS or tandem MS detection systems [23]. Indeed, several drawbacks, such as the bulky size and real cost to acquire and regularly maintain LC-MS instruments, and environmental conditions in the laboratory that need to be well controlled to guarantee system stability, as well as the need for highly educated, trained, and skilled operators, have prevented the analysis of eCB content as a routine. Needless to say, high throughput eCB quantitation for clinical diagnosis of human diseases remains as yet far from reach.

Against this background, the present data seem to provide a proof of concept that a rapid, efficient, user friendly and inexpensive quantitation of eCBs in biological matrices is indeed feasible, pointing to the new BIONOTE device as a valuable prototype to be further implemented to reach the sensitivity of classic LC/MS instruments.

## Data Availability

Data available on request due to ethical restrictions.

## References

[1] Devane W.A., Hanus L., Breuer A., Pertwee R.G., Stevenson L.A., Griffin G., Gibson D., Mandelbaum A., Etinger A., Mechoulam R. (1992). Isolation and structure of a brain constituent that binds to the cannabinoid receptor. Science.

[2] Mechoulam R., Ben-Shabat S., Hanus L., Ligumsky M., Kaminski N.E., Schatz A.R., Gopher A., Almog S., Martin B.R., Compton D.R. (1995). Identification of an endogenous 2-monoglyceride, present in canine gut, that binds to cannabinoid receptors. Biochem. Pharmacol..

[3] Sugiura T., Kondo S., Sukagawa A., Nakane S., Shinoda A., Itoh K., Yamashita A., Waku K. (1995). 2-Arachidonoylgylcerol: A possible endogenous cannabinoid receptor ligand in brain. Biochem. Biophys. Res. Commun..

[4] Matsuda L.A., Lolait S.J., Brownstein M.J., Young A.C., Bonner T.I. (1990). Structure of a cannabinoid receptor and functional expression of the cloned cDNA. Nature.

[5] Munro S., Thomas K.L., Abu-Shaar M. (1993). Molecular characterization of a peripheral receptor for cannabinoids. Nature.

[6] Di Marzo V., Fontana A. (1995). Anandamide, an endogenous cannabinomimetic eicosanoid: ‘killing two birds with one stone’. Prostaglandins Leukot. Essent. Fat. Acids.

[7] Okamoto Y., Morishita J., Tsuboi K., Tonai T., Ueda N. (2004). Molecular characterization of a phospholipase D generating anandamide and its congeners. J. Biol. Chem..

[8] Bisogno T., Howell F., Williams G., Minassi A., Cascio M.G., Ligresti A., Matias I., Schiano-Moriello A., Paul P., Williams E.J. (2003). Cloning of the first sn1-DAG lipases points to the spatial and temporal regulation of endocannabinoid signaling in the brain. J. Cell Biol..

[9] Cravatt B., Giang D.K., Mayfield S.P., Boger D.L., Lerner R.A., Gilula N.B. (1996). Molecular characterization of an enzyme that degrades neuromodulatory fatty-acid amides. Nature.

[10] Dinh T.P., Carpenter D., Leslie F.M., Freund T.F., Katona I., Sensi S.L., Kathuria S., Piomelli D. (2002). Brain monoglyceride lipase participating in endocannabinoid inactivation. Proc. Natl. Acad. Sci. USA.

[11] Maccarrone M. (2020). Missing pieces to the endocannabinoid puzzle. Trends Mol. Med..

[12] Maccarrone M., Bab I., Bíró T., Cabral G.A., Dey S.K., Di Marzo V., Konje J.C., Kunos G., Mechoulam R., Pacher P. (2015). Endocannabinoid signaling at the periphery: 50 years after THC. Trends Pharmacol. Sci..

[13] Friedman D., French J.A., Maccarrone M. (2019). Safety, efficacy, and mechanisms of action of cannabinoids in neurological disorders. Lancet Neurol..

[14] Matias I., Gatta-Cherifi B., Tabarin A., Clark S., Leste-Lasserre T., Marsicano G., Piazza P.V., Cota D. (2012). Endocannabinoids measurement in human saliva as potential biomarker of obesity. PLoS ONE.

[15] Lafreniere J.D., Lehmann C. (2017). Parameters of the endocannabinoid system as novel biomarkers in sepsis and septic shock. Metabolite.

[16] Fanelli F., Mezzullo M., Repaci A., Belluomo I., Gasparini D.I., Di Dalmazi G., Mastroroberto M., Vicennati V., Gambineri A., Morselli-Labate A.M. (2018). Profiling plasma N–acylethanolamine levels and their ratios as a biomarker of obesity and dysmetabolism. Mol. Metab..

[17] Azar S., Sherf-Dagan S., Nemirovski A., Webb M., Raziel A., Keidar A., Goitein D., Sakran N., Shibolet O., Tam J. (2019). Circulating endocannabinoids are reduced following bariatric surgery and associated with improved metabolic homeostasis in humans. Obes. Surg..

[18] Aran A., Eylon M., Harel M., Polianski L., Nemirovski A., Tepper S., Schnapp A., Cassuto H., Wattad N., Tam J. (2019). Lower circulating endocannabinoid levels in children with autism spectrum disorder. Mol. Autism.

[19] Cecconi S., Rapino C., Di Nisio V., Rossi G., Maccarrone M. (2020). The (endo) cannabinoid signaling in female reproduction: What are the latest advances?. Prog. Lipid Res..

[20] Cristino L., Bisogno T., Di Marzo V. (2020). Cannabinoids and the expanded endocannabinoid system in neurological disorders. Nature Rev. Neurol..

[21] Mallipedhi A., Prior S.L., Dunseath G., Bracken R.M., Barry J., Caplin S. (2015). Changes in plasma levels of.arachidonoyl ethanolamine and N–palmitoylethanolamine following bariatric surgery in morbidly obese females with impaired glucose homeostasis. J. Diabetes Res..

[22] Wang X., Yu Q., Yue H., Zhang J., Zeng S., Cui F. (2016). Circulating endocannabinoids and insulin resistance in patients with obstructive sleep apnea. BioMed Res. Int..

[23] Marchioni C., de Souza I.D., Junior V.R.A., de Souza Crippa J.A., Tumas V., Queiroz M.E.C. (2018). Recent advances in LC-MS/MS methods to determine endocannabinoids in biological samples: Application in neurodegenerative diseases. Anal. Chim. Acta.

[24] Damborský P., Švitel J., Katrlík J. (2016). Optical biosensors. Essays Biochem..

[25] Chen Y., Ren R., Pu H., Chang J., Mao S., Chen J. (2017). Field-effect transistor biosensors with two-dimensional black phosphorus nanosheets. Biosens. Bioelectron..

[26] Fu Y.Q., Luo J.K., Nguyen N.T., Walton A.J., Flewitt A.J., Zu X.T., Li Y., McHale G., Matthews A., Iborra E. (2017). Advances in piezoelectric thin films for acoustic biosensors, acoustofluidics and lab-on-chip applications. Prog. Mat. Sci..

[27] Pohanka M. (2018). Overview of piezoelectric biosensors, immunosensors and DNA sensors and their applications. Materials.

[28] Ertürk G., Mattiasson B. (2017). Capacitive biosensors and molecularly imprinted electrodes. Sensors.

[29] Stomelli V., Leoni A., Ferri G., Errico V., Ricci M., Pallotti A., Saggio G. A multi-source energy harvesting sensory glove electronic architecture. Proceedings of the 3rd International Conference on Smart and Sustainable Technologies (SpliTech).

[30] Hertel F., Li S., Chen M., Pott L., Mehta S., Zhang J. (2019). Fluorescent biosensors for multiplexed imaging of phosphoinositide dynamics. ACS Chem. Biol..

[31] Justino C.I., Duarte A.C., Rocha-Santos T.A. (2016). Critical overview on the application of sensors and biosensors for clinical analysis. TrAC Trends Anal. Chem..

[32] Skrzypiec M., Weiss M., Dopierała K., Prochaska K. (2019). Langmuir-Blodgett films of membrane lipid in the presence of hybrid silsesquioxane, a promising component of biomaterials. Mat. Sci. Eng. C.

[33] Bussetti G., Violante A., Yivlialin R., Cirilli S., Bonanni B., Chiaradia P., Goletti C., Tortora L., Paolesse R., Martinelli E. (2011). Site-sensitive gas sensing and analyte discrimination in langmuir—Blodgett porphyrin films. J. Phys. Chem. C.

[34] D’Amico A., Di Natale C., Falconi C., Pennazza G., Santonico M., Lundstrom I. (2017). Equivalent electric circuits for chemical sensors in the langmuir regime. Sens. Actuators B Chem..

[35] Santonico M., Pennazza G., Grasso S., D’Amico A., Bizzarri M. (2013). Design and test of a biosensor-based multisensorial system: A proof of concept study. Sensors.

[36] Capuano R., Santonico M., Pennazza G., Ghezzi S., Martinelli E., Roscioni C., Lucantoni G., Galluccio G., Paolesse R., Di Natale C. (2015). The lung cancer breath signature: A comparative analysis of exhaled breath and air sampled from inside the lungs. Sci. Rep..

[37] Scarlata S., Pennazza G., Santonico M., Santangelo S., Bartoli I.R., Rivera C., Vernile C., De Vincentis A., Incalzi R.A. (2017). Screening of obstructive sleep apnea syndrome by electronic-nose analysis of volatile organic compounds. Sci. Rep..

[38] Oddi S., Fezza F., Pasquariello N., D’Agostino A., Catanzaro G., De Simone C., Rapino C., Finazzi-Agrò A., Maccarrone M. (2009). Molecular identification of albumin and Hsp70 as cytosolic anandamide-binding proteins. Chem. Biol..

[39] Baggelaar M.P., Maccarrone M., van der Stelt M. (2018). 2-Arachidonoylglycerol: A signaling lipid with manifold actions in the brain. Prog. Lipid Res..

[40] Grasso S., Santonico M., Bisogno T., Pennazza G., Zompanti A., Sabatini A., Maccarrone M. (2018). An innovative liquid biosensor for the detection of lipid molecules involved in diseases of the nervous system. Multidiscip. Digit. Publ. Inst. Proc..

[41] Xiong X., Zhang L., Cheng L., Mao W. (2015). High-throughput salting-out assisted liquid–liquid extraction with acetonitrile for the determination of anandamide in plasma of hemodialysis patients with liquid chromatography tandem mass spectrometry. Biomed. Chromatogr..

[42] Mwanza C., Chen Z., Zhang Q., Chen S., Wang W., Deng H. (2016). Simultaneous HPLC-APCI-MS/MS quantification of endogenous cannabinoids and glucocorticoids in hair. J. Chromatogr. B.

[43] Sergi M., Battista N., Montesano C., Curini R., Maccarrone M., Compagnone D. (2013). Determination of the two major endocannabinoids in human plasma by μ-SPE followed by HPLC-MS/MS. Anal. Bioanal. Chem..

[44] Ji D., Jang C.G., Lee S. (2014). A sensitive and accurate quantitative method to determine N-arachidonoyldopamine and N-oleoyldopamine in the mouse striatum using column-switching LC–MS–MS: Use of a surrogate matrix to quantify endogenous compounds. Anal. Bioanal. Chem..

